# Breakthrough reactions in pediatric chemotherapeutic desensitization: Outcomes and associated risk factors

**DOI:** 10.1111/pai.70425

**Published:** 2026-07-13

**Authors:** Pathitta Doradee, Supaluk Tangvalelerd, Kantima Kanchanapoomi, Prapasri Kulalert, Punchama Pacharn, Orathai Jirapongsananuruk, Witchaya Srisuwatchari

**Affiliations:** ^1^ Division of Allergy and Immunology, Department of Pediatrics, Faculty of Medicine Siriraj Hospital Mahidol University Bangkok Thailand; ^2^ Department of Clinical Epidemiology, Faculty of Medicine Thammasat University Pathum Thani Thailand; ^3^ Division of Allergy and Immunology, Department of Pediatrics, Faculty of Medicine Thammasat University Pathum Thani Thailand

**Keywords:** breakthrough reaction, chemotherapy, hypersensitivity reactions, pediatrics, rapid drug desensitization, risk factors

## Abstract

**Background:**

Rapid drug desensitization (RDD) enables the safe reintroduction of chemotherapeutic agents in patients with hypersensitivity reactions (HSRs). However, some patients experience breakthrough reactions (BTRs) during desensitization, and research on factors associated with BTRs is limited in pediatric patients.

**Methods:**

We conducted an ambispective observational cross‐sectional study in Bangkok, Thailand. Patients ≤18‐year‐old who experienced immediate HSRs (within 1–6 h) to chemotherapeutic agents and underwent RDD following evaluation by allergists between January 2010 and April 2025 were included.

**Results:**

A total of 182 RDDs were performed in 38 children (median age of 5.3 years). The majority were diagnosed with acute leukemia (47.4%), and the most common causative agents for HSRs were *E. coli*‐Asparaginase (26.9%), vincristine (24.7%), and methotrexate (13.7%). Asparaginase, vincristine, cyclophosphamide, carboplatin/oxaliplatin, and doxorubicin were commonly associated with Type I HSRs, while methotrexate, cyclosporin A and etoposide were linked to Type I HSRs as well as infusion‐related reactions (IRRs) or cytokine‐release reactions (CRRs). Twenty‐nine BTRs occurred during RDD (15.9%), most of which were mild (72.4%). Independent risk factors for BTRs included *E. coli*‐Asparaginase desensitization (adjusted OR 3.87 [1.45, 10.39], *p* = .007), and a higher initial starting dose (ratio ≤1:10,000) (adjusted OR 3.51 [1.02, 12.18], *p* = .048). Conversely, later cycles of desensitization reduced the risk of BTRs (adjusted OR 0.47 [0.31, 0.72], *p* < .001).

**Conclusion:**

RDDs for chemotherapeutic agents effectively enable safe treatment continuation in most children with immediate HSRs, including type I HSR, CRR, and mixed reactions. In our study, *E. coli*‐Asparaginase, a higher initial starting dose, and an early desensitization cycle were identified as having potential risk factors associated with BTRs.

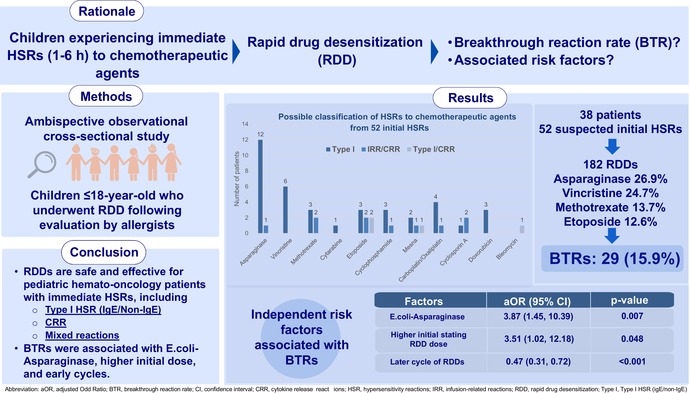

## Graphical abstract

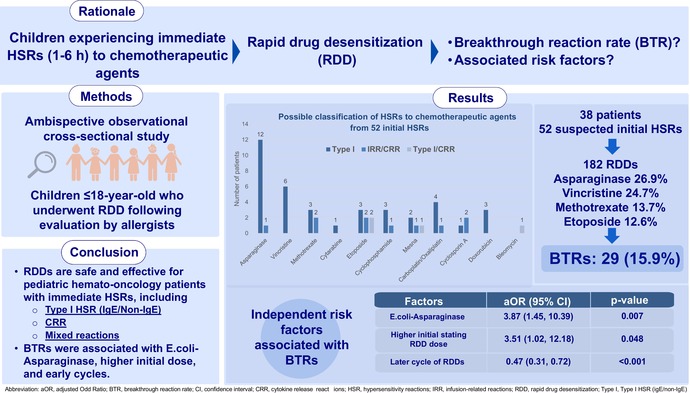

**Key message**

Rapid drug desensitization (RDD) allows safe reintroduction of chemotherapy in children with immediate hypersensitivity reactions (HSR), including type I HSR (IgE and non‐IgE), cytokine‐release reactions, and mixed reactions. This study identified *E. coli*‐Asparaginase, higher initial doses, and early desensitization cycles as potential risk factors for breakthrough reactions during RDD, emphasizing the need for careful consideration of these factors to optimize safety.

## 1 INTRODUCTION

The use of chemotherapeutic agents has expanded and remains the mainstay of cancer treatment in both adults and children, including for common pediatric cancers such as hematologic malignancies and solid tumors. However, hypersensitivity reactions (HSRs) to these agents are increasingly observes in both groups.^1^, ^2^, ^3^, ^4^, ^5^ Reported risk factors—personal allergic disease, prior drug HSRs, and mastocytosis—are inconsistent across studies and may differ between adults and children.^5^, ^6^, ^7^ In contrast, repeated exposure and higher cumulative doses of platinum‐based compounds are consistently associated with a higher risk of HSRs.^5^, ^8^ These HSRs are unpredictable, ranging from mild symptoms to life‐threatening anaphylaxis,^9^, ^10^ and can force delays, discontinuation, or substitution of first‐line therapy, potentially worsening outcomes.^1^

Rapid drug desensitization (RDD) is a key strategy to permit safe reexposure to culprit agents, primarily for type I HSRs, and also used for certain cytokine‐release syndrome, mixed reactions, and type IV HSRs—excluding severe cutaneous adverse reactions (SCARs).^1^, ^3^, ^5^, ^11^, ^12^ RDD induces temporary tolerance by administering progressively increasing doses over a short period until the cumulative therapeutic dose is achieved.^3^, ^13^

Although RDD is generally well tolerated, breakthrough reactions (BTRs)—HSRs occurring during desensitization^13^—reported in approximately 9%–25% of patients. While many BTRs are mild, moderate to severe events can interrupt protocols.^6^, ^14^, ^15^, ^16^, ^17^ Clinicians therefore individualize subsequent RDD regimens with measures such as enhanced premedication strategies, slower dose escalation, lower starting concentrations, or temporary dose reductions.^1^

Adult studies have identified potential BTRs predictors, including the severity of the initial HSR,^14^, ^15^, ^16^ frequency of prior chemotherapy exposure,^14^, ^16^ prior drug HSR^16^ or atopy,^6^ positive skin test,^6^, ^17^ elevated total IgE levels (≥100 IU/mL),^14^ and corticosteroid premedication^15^—mostly in platinum‐based agents, taxanes, or biologic agents. Pediatric data—especially in hemato‐oncology—are limited,^2^, ^18^, ^19^ and pediatric‐specific predictors remain unclear. A more comprehensive understanding of the incidence, clinical features, and predictive factors for BTRs in pediatric patients may support safer and more standardized implementation of RDD protocols in pediatric oncology. Therefore, this study aims to characterize BTRs occurring during chemotherapeutic desensitization in children with immediate HSRs, with a particular focus on clinical outcomes and associated risk factors.

## 2 METHODS

We conducted an ambispective observational cross‐sectional study. Pediatric patients aged ≤18 years who experienced immediate hypersensitivity reactions (IHRs)^3^—defined as occurring within 1–6 h after receiving the latest dose of the index chemotherapeutic agents or systemic protective agent against chemotherapy toxicity (e.g., sodium‐2‐mercaptoethanesulfonate, Mesna)—and who underwent desensitization at the Department of Pediatrics, Siriraj Hospital Mahidol University, were consecutively included. The study included a retrospective period (January 1st 2010–March 6th 2024) and a prospective period (March 7th 2024–April 30th 2025). Patients with incomplete medical records were excluded from the study. The study was approved by the Institutional Review Board of Siriraj Hospital, Mahidol University (Certificate of Approval No. Si 183/2024). Written informed consent was obtained from participants or their parents for participation in the prospective study.

### 2.1 Desensitization procedures

Candidates for RDD included patients with type I (IgE and non‐IgE), cytokine‐release reactions (CRR), infusion‐related reactions (IRR), or mixed‐type reactions when continued use of the implicated agent was clinically indicated and the reaction was not a contraindication (e.g., severe cutaneous adverse reactions). All HSRs and desensitization procedures were evaluated and conducted under the supervision of pediatric allergists. These procedures were performed in a fully equipped pediatric ICU or in a unit capable of closely monitoring, recognizing, and treating allergic reactions if they occurred. Desensitization protocols were individualized based on initial reaction severity, culprit agent, and prior desensitization history. Typically, initiation dose ranged from 1/1,000,000 to 1/10,000 of the therapeutic dose. Doses were then doubled at 15‐min intervals using multiple infusion bags until the target therapeutic dose was reached; occasional exceptions were made for final steps as clinically required.^13^ Premedication was applied based on the phenotype and clinical features of the index reaction. Options included antihistamines for cutaneous/gastrointestinal symptoms, leukotriene receptor antagonists (e.g., montelukast) to prevent bronchospasm when indicated, and systemic corticosteroids for systemic symptoms (e.g., severe chills, rigors, fever, or pain). In some cases, these agents were already part of the standard chemotherapy premedication regimen.

### 2.2 Data collection

The following data were extracted from participants' electronic medical records: demographic information, personal and family history of allergic diseases, principal diagnosis, clinical manifestations, associated symptoms, and the severity of initial HSRs, which were classified according to Brown's grading system,^9^ Additionally, information regarding whether the initial reaction occurred during the first or subsequent infusion, the treatment provided during initial reactions, and details of the desensitization protocol were also collected.

### 2.3 Breakthrough reactions (BTRs)

BTRs were defined as HSR that occurred during the desensitization procedure despite adherence to the protocol and premedication.^13^ Data on BTR occurrence rate, severity, treatment during BTR, and the completion status of desensitization following BTR were also collected. Complete (successful) desensitization was defined as reaching the full therapeutic dose after any necessary treatment for a BTR and tolerating subsequent administrations. Incomplete desensitization referred to termination of the RDD before the target dose was achieved due to persistent or severe reactions.^13^

### 2.4 Serum tryptase measurement

Serum tryptase levels were measured using the ImmunoCAP™ (Thermo Fisher Scientific, Uppsala, Sweden) in selected cases with diagnostic uncertainty with anaphylaxis. The assay had the lowest limit of detection of ≤0.1 μg/L. When available, paired measurements were taken during acute events within 2 h after the onset of symptoms and at a baseline time point at least 24 h after all signs and symptoms had resolved.

### 2.5 Drug allergy investigation

In some patients, an allergy work‐up was conducted after desensitization to identify the culprit drug. Drug allergy work‐ups followed the guidelines established by the European Network on Drug Allergy (ENDA) and the Drug Allergy Interest Group of the European Academy of Allergy and Clinical Immunology (EAACI). The stepwise approach included skin testing (ST)—starting with a skin prick test (SPT), followed by an intradermal test (IDT) if the SPT was negative—and drug provocation tests (DPT).^3^, ^20^ For SPT and IDT, the intravenous formulation of the suspected drug at the recommended nonirritating concentration was used.^5^, ^21^ Patients were classified as having a confirmed drug HSR if they experienced a BTR or had a positive allergy test, including a positive ST or a positive DPT. However, some patients remained ineligible for diagnostic testing due to clinical instability, recent antihistamine use, or severe reactions within the past 4–6 weeks.

### 2.6 Phenotyping and endotyping of initial hypersensitivity reactions

We categorized patients' initial HSRs into the following phenotypes/endotypes: type I (IgE‐mediated and non‐IgE‐mediated reactions), CRR, IRR, and mixed‐type reactions.^22^, ^23^ Classification was based primarily on a thorough review of their clinical history, with CRRs and IRRs further distinguished by clinical behavior and response to premedication—IRRs tend to be self‐limiting on repeated exposure and typically respond to premedication.

Type I HSRs (IgE/Non‐IgE) often occurred with repeated infusions. Clinical manifestations range from mild symptoms (flushing, pruritus, urticaria) to respiratory symptoms (dyspnea, wheeze), hypotension, and life‐threatening anaphylaxis. Supportive diagnostic evidence for IgE‐mediated mechanisms includes elevated serum tryptase and positive ST and/or DPT.

CRRs often occur during initial exposures but are not limited to first infusions. Typical features include flushing, chills, fever, headache, tachycardia, nausea, and syncope. CRRs frequently do not improve with standard premedication or slower infusion rates during the initial exposure. IRRs may also present during early infusions but tend to be milder and self‐limiting symptoms*—*such as flushing, tachycardia, and nausea*—*that often improve with premedication or on repeated exposure. Mixed‐type reactions reflect overlapping mechanisms and clinical features of both type I (IgE‐ or non‐IgE‐mediated) HSRs and cytokine‐release responses.^22^, ^23^

### 2.7 Statistical analysis

All statistical analyses were performed using SPSS version 25.0 (SPSS, Inc., Chicago, IL, USA) and Stata version 17 (StataCorp LP, College Station, TX, USA). Categorical data are presented as numbers and percentages and were analyzed using the Chi‐squared or Fisher's exact test, as appropriate. Continuous variables (nonnormally distributed) are presented as the median and interquartile range (IQR) and were analyzed using the Mann–Whitney *U* test. A *p*‐value less than 0.05 was considered statistically significant. Univariate and multivariate logistic regression analyses were conducted to identify risk factors associated with BTRs. Variables with a *p*‐value <0.05 in the univariate analysis were considered candidates for the multivariable logistic regression model. The results of the analysis are presented as odds ratios (ORs) with 95% confidence intervals (CIs) for both univariable and multivariable logistic regression analyses.

## 3 RESULTS

### 3.1 Patient characteristics and clinical manifestation during index hypersensitivity reactions

We included 38 pediatric patients with 52 suspected initial HSRs (Table 1). Median age at index HSRs was 5.3 years (IQR 3.5, 12.1 years), 63.2% were male. Primary diagnoses were acute leukemia (47.4%), various solid tumors (42.1%), lymphoma (5.3%), aplastic anemia (2.6%) and Langerhans cell histiocytosis (2.6%). Family or personal histories of allergic diseases was uncommon (5.3% each), and 26.3% had prior drug HSRs.

**TABLE 1:** Clinical characteristics of study participants and clinical manifestations of the index hypersensitivity reactions (HSRs).

Study participant characteristics and index HSRs manifestation	Total patients (*N* = 38)
Age at index HSRs (years), median (IQR)	5.3 (3.5, 12.1)
Male, *n* (%)	24 (63.2%)
Personal allergic diseases	
Allergic rhinitis, *n* (%)	2 (5.3%)
Family history of allergic diseases, *n* (%)	2 (5.3%)
Principal diagnosis, *n* (%)	
Acute leukemia	18 (47.4%)
Solid tumors^a^	16 (42.1%)
Lymphoma	2 (5.3%)
Aplastic anemia	1 (2.6%)
Langerhans cell histiocytosis	1 (2.6%)
History of other drug HSRs, *n* (%)	10 (26.3%)
Onset of index HSRs (minutes), median (IQR)^b^	20 (1.3, 80)
Clinical manifestation during index HSRs, *n* (%)	
Mucocutaneous	37 (97.4%)
Gastrointestinal	15 (39.5%)
Respiratory	11 (28.9%)
Cardiovascular	4 (10.5%)
Neurological	3 (7.9%)
Associated symptoms, *n* (%)	
Fever	1 (2.6%)
Diaphoresis	2 (5.3%)
Baseline serum tryptase level (μg/L), median (IQR)^c^	1.73 (1.21, 2.56)
Acute serum tryptase level (μg/L), median (IQR)^c^	2.80 (1.39, 6.85)
Severity of index HSRs according to Brown's grading system, *n* (%)	
Mild	19 (50%)
Moderate	15 (39.5%)
Severe	4 (10.5%)

Abbreviations: HSR, hypersensitivity reactions; IQR, interquartile range.

^a^ Solid tumors (*n* = 16), for example, germ cell tumor (*n* = 4), neuroblastoma (*n* = 4), osteosarcoma (*n* = 3), retinoblastoma (*n* = 2), colorectal adenocarcinoma (*n* = 1), Ewing sarcoma (*n* = 1), hepatoblastoma (*n* = 1).

^b^ From the initial 52 suspected drugs.

^c^ Data are available for 9 patients. In an additional 2 patients, levels were ≤0.1 μg/L (the lower limit of detection) and are therefore not included in the table.

**FIGURE 1:**
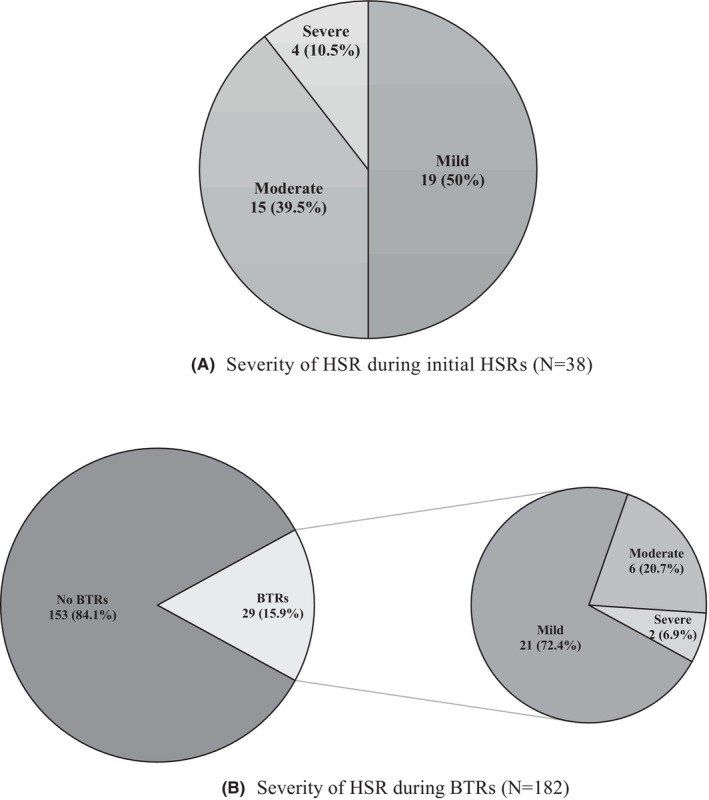
Severity of hypersensitivity reactions (HSR) according to Brown's grading system: (A) during initial HSRs and (B) during breakthrough reactions.

The median onset of symptoms following the last dose chemotherapeutic administration was 20 min (IQR 1.3, 80 min). Mucocutaneous manifestations predominated (97.4%), followed by gastrointestinal symptoms (39.5%), respiratory (28.9%), cardiovascular symptoms (10.5%), and neurological symptoms (7.9%). According to Browns' grading system, index HSRs were 50% mild, 39.5% moderate, and 10.5% severe (Figure 1A).

Serum tryptase was available for 11 patients: two values were below the assay detection limit (≤0.1 μg/L). Among the remaining nine, median baseline tryptase was 1.73 μg/L (IQR 1.21, 2.56 μg/L) and median acute tryptase was 2.80 μg/L (IQR 1.39, 6.85 μg/L).

### 3.2 Possible classification of hypersensitivity reactions to chemotherapy

We performed phenotyping/endotyping for the 52 suspected initial HSRs; detailed case‐level data and the full classification are provided in Tables S1 and S2. In summary, most reactions were classified as Type I HSRs (73.1%), with smaller proportions of IRRs (11.5%), CRRs (7.7%), and mixed‐type reactions (7.7%). Clinically, type I HSRs were most commonly associated with asparaginase, vincristine, cyclophosphamide, carboplatin/oxaliplatin, and doxorubicin, whereas methotrexate, cyclosporin A, and etoposide were associated to both type I and IRR/CRR patterns.

### 3.3 Chemotherapeutic rapid drug desensitization and breakthrough reactions

A total of 182 RDD procedures to 11 chemotherapeutic agents were performed in 38 patients (Table 2). BTRs occurred in 29 RDDs (15.9%); by Brown's grading these were predominantly mild (72.4%), with 20.7% moderate, and 6.9% severe (Figure 1B). By phenotype, 42.1% of patients with type I HSR experienced BTR during RDD; no BTRs were observed among patients with IRR or mixed‐type reactions in this cohort, whereas all four patients with CRR experienced BTR.

**TABLE 2:** Clinical characteristics during chemotherapeutic rapid drug desensitization (RDD) compared between patients with and without breakthrough reactions (BTR).

	Total (*N* = 182)	BTR (*n* = 29)	No BTR (*n* = 153)	*p*
Age at RDD (years), median (IQR)	5.7 (4.2, 12.1)	5.7 (3.9, 7.8)	5.7 (4.3, 13.0)	.162
Chemotherapeutic drugs, *n* (%)				
*E. coli*‐Asparaginase	49 (26.9%)	16 (55.2%)	33 (21.6%)	**<.001**
Other chemotherapeutic drugs				
Vincristine	45 (24.7%)	0	45 (29.4%)	
Methotrexate	25 (13.7%)	4 (13.8%)	21 (13.7%)	
Etoposide	23 (12.6%)	0	23 (15%)	
Cyclophosphamide	7 (3.9%)	1 (3.4%)	6 (3.9%)	
Platinum	13 (7.1%)	2 (6.9%)	11 (7.2%)	
Cyclosporin A	7 (3.9%)	3 (10.3%)	4 (2.6%)	
Sodium‐2‐mercaptoethanesulfonate (Mesna)	7 (3.9%)	2 (6.9%)	5 (3.3%)	
Doxorubicin	3 (1.6%)	1 (3.4%)	2 (1.3%)	
Bleomycin	2 (1.1%)	0	2 (1.3%)	
Cytarabine	1 (0.6%)	0	1 (0.7%)	
Initial starting ratio				
1:10,000 of total therapeutic dose (higher initial dose)	15 (8.2%)	7 (24.1%)	8 (5.2%)	.**003**
1:10,000 of total therapeutic dose (lower initial dose)	167 (91.8%)	22 (75.9%)	145 (94.8%)	
No. of bags				
2 bags	6 (3.3%)	3 (10.3%)	3 (2.0%)	.144
3 bags	83 (45.6%)	12 (41.4%)	71 (46.4%)	
4 bags	81 (44.5%)	12 (41.4%)	69 (45.1%)	
5 bags	12 (6.6%)	2 (6.9%)	10 (6.5%)	
Premedication before RDD				
H1 antihistamines	79 (43.4%)	19 (65.5%)	60 (39.2%)	.**009**
H2 antihistamines	58 (31.9%)	14 (48.3%)	44 (28.8%)	.**039**
Systemic corticosteroid	63 (34.6%)	10 (34.5%)	53 (34.6%)	.987
Leukotriene receptor antagonist	16 (8.8%)	6 (20.7%)	10 (6.5%)	.**025**
Desensitization cycle no.	3 (1.6)	1 (1.2)	3 (2.6)	**<.001**
Status of confirmation of the culprit drug each time before RDD	62 (34.1%)	13 (44.8%)	49 (32%)	.182

*Note*: Bolded values indicate statistically significant differences between groups.

Abbreviation: BTR, breakthrough reaction; HSR, hypersensitivity reactions; IQR, interquartile range; RDD, rapid drug desensitization.

**FIGURE 2:**
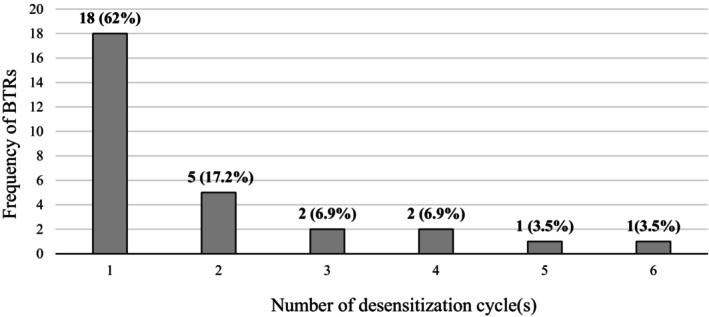
Frequency of breakthrough reactions (BTRs) stratified by the number of desensitization cycles.

BTRs were significantly more frequent with *E. coli*‐Asparaginase than with other agents (55.2% vs. 21.6%, *p* < .001). RDDs initiated at a lower starting ratio (≤1:10,000 of the total therapeutic dose, corresponding to a higher initial dose) also had a higher BTR frequency (24.1% vs. 5.2%, *p* = .003). BTRs were more common in earlier desensitization cycles (median cycle 1 [IQR 1, 2] vs. cycles 3 [IQR 2, 6], *p* < .001); the cycles‐specific frequency is shown in Figure 2. Premedications—including H1‐antihistamines, H2‐antihistamines, and leukotriene receptor antagonist (LTRA)—were used more often in patients experiencing BTR. There were no statistically significant differences between RDDs with and without BTR regarding patient age at RDD, number of infusion bags, use of systemic corticosteroids premedication, or confirmation status of the culprit drug prior to RDD (all *p* > .05).

### 3.4 Characteristics of patients with breakthrough reactions

Among 29 BTRs, 19 procedures (65.5%) were completed after treatment, and 10 (34.5%) were terminated before reaching the target dose (Table 3). Compared to completed RDDs, incomplete RDDs were significantly associated with respiratory manifestations (40% vs. 5.3%, *p* = .036), *E. coli*‐asparaginase as the implicated agent (90% vs. 36.8%, *p* = .008), and intramuscular adrenaline use (50% vs. 5.3%, *p* = .011). There was no overall significant difference in BTR severity distribution by Brown's grading (*p* = .064), although severe reactions were observed only in the incomplete RDD group.

**TABLE 3:** Clinical characteristics of patients with breakthrough reactions (BTR) during chemotherapeutic rapid drug desensitization (RDD), compared between patients with complete and incomplete RDD.

	Total BTR (*n* = 29)	BTR, complete RDD (*n* = 19)	BTR, incomplete RDD (*n* = 10)	*p*
Age at RDD (years), median (IQR)	5.7 (3.9,7.8)	6.0 (4.3,11.9)	4.1 (1.2,7.3)	.241
Clinical manifestation during BTRs, *n* (%)				
Mucocutaneous	26 (89.7%)	18 (94.7%)	8 (80%)	.267
Gastrointestinal	6 (20.7%)	3 (15.8%)	3 (30%)	.633
Respiratory	5 (17.2%)	1 (5.3%)	4 (40%)	.**036**
Neurological	0	0	0	‐
Cardiovascular	0	0	0	‐
Associated symptoms				
Fever	0	0	0	‐
Diaphoresis	1 (3.5%)	1 (5.3%)	0	1.000
Severity of BTRs according to Brown's grading system, *n* (%)				
Mild	21 (72.4%)	16 (84.2%)	5 (50%)	.064
Moderate	6 (20.7%)	3 (15.8%)	3 (30%)	
Severe	2 (6.9%)	0	2 (20%)	
Chemotherapeutic drugs, *n* (%)				
*E*. coli‐Asparaginase	16 (55.2%)	7 (36.8%)	9 (90%)	.**008**
Methotrexate	4 (13.8%)	4 (21.1%)	0	.268
Cyclosporin A	3 (10.3%)	3 (15.8%)	0	.532
Sodium‐2‐mercaptoethanesulfonate (Mesna)	2 (6.9%)	2 (10.5%)	0	.532
Platinum	2 (6.9%)	2 (10.5%)	0	.532
Cyclophosphamide	1 (3.4%)	1 (5.3%)	0	1.000
Doxorubicin	1 (3.4%)	0	1 (10%)	.345
Duration of RDD (hours), median (IQR)	6.8 (4.8, 9.5)	6.5 (4.0,7.8)	8.4 (5.2,10.5)	.261
No. of bags				
2 bags	3 (10.3%)	2 (10.5%)	1 (10%)	.365
3 bags	12 (41.4%)	10 (52.6%)	2 (20%)	
4 bags	12 (41.4%)	6 (31.6%)	6 (60%)	
5 bags	2 (6.9%)	1 (5.3%)	1 (10%)	
Treatment during BTRs				
Adrenaline IM	6 (20.7%)	1 (5.3%)	5 (50%)	.**011**
H1 antihistamines	25 (86.2%)	16 (84.2%)	9 (90%)	1.000
H2 antihistamines	14 (48.3%)	8 (42.1%)	6 (60%)	.450
Systemic corticosteroid	5 (17.2%)	3 (15.8%)	2 (20%)	1.000
Leukotriene receptor antagonist	3 (10.3%)	2 (10.5%)	1 (10%)	1.000

*Note*: Bolded values indicate statistically significant differences between groups.

Abbreviation: BTR, breakthrough reaction; HSR, hypersensitivity reactions; IQR, interquartile range; RDD, rapid drug desensitization; IM, intramuscular route of administration.

We reviewed the clinical circumstances leading to termination of RDD in the 10 incomplete procedures among 6 patients. Five procedures were terminated because patients developed moderate to severe reactions that required intramuscular adrenaline (all five were associated with *E. coli*‐asparaginase). The remaining five incomplete procedures involved predominantly mild cutaneous symptoms (urticaria or angioedema)—four with *E. coli*‐asparaginase and one with doxorubicin—that persisted or recurred despite premedication (H1/H2 antihistamines in all five; four also received systemic corticosteroids), prompting termination for safety.

Follow‐up actions after incomplete RDD included: switching from *E. coli*‐asparaginase to Erwinia asparaginase in one patient; repeating RDD to *E. coli*‐asparaginase in three patients with successful subsequent completion at later cycles; and changing the chemotherapy regimen to omit the culprit agent in two patients (one *E. coli*‐asparaginase, one doxorubicin).

### 3.5 Confirming of culprit drug casing hypersensitivity reaction

The confirmation test results from 38 patients with 52 suspected HSRs revealed confirmed culprit drugs in 20 of these patients (52.6%). This finding corresponds to 24 chemotherapeutic agents (46.2%) identified from the 52 suspected drugs, as shown in Table S3.

### 3.6 Potential risk factors associated with breakthrough reactions

Logistic regression identified several factors associated with BTRs; full univariable results are presented in Table 4. In multivariable analysis, three variables remained independently associated with BTRs during RDD: *E. coli*‐asparaginase exposure (adjusted OR 3.87; 95% CI 1.45, 10.39; *p* = .007) and an initial starting ratio ≤1:10,000 of the total therapeutic dose (higher initial dose) (adjusted OR 3.51; 95% CI 1.02, 12.18; *p* = .048) were associated with increased BTR risk. By contrast, a greater number of desensitization cycles was protective; each additional cycle reduced the odds of BTR (adjusted OR 0.47; 95% CI 0.31, 0.72; *p* < .001). Although premedication with H1/H2 antihistamines and leukotriene receptor antagonists was associated with BTRs in univariable analysis, these associations did not persist after adjustment.

**TABLE 4:** Univariable and multivariable logistic regression analysis to identify associated prognostic factors of breakthrough reactions during rapid drug desensitization.

Potential risk factors	Univariable logistic regression			Multivariable logistic regression		
	Crude OR	95% CI	*p*	Adjusted OR	95% CI	*p*
Chemotherapeutic drugs						
*E. coli*‐Asparaginase	4.48	1.96, 10.23	**<.001**	3.87	1.45, 10.39	.**007**
Other chemotherapeutic drugs	1					
Initial starting ratio						
**≤**1:10,000 of total therapeutic dose (higher initial dose)	5.77	1.90, 17.48	.**002**	3.51	1.02, 12.18	.**048**
>1:10,000 of total therapeutic dose (lower initial dose)	1					
Desensitization cycle no.	0.59	0.43, 0.82	.**001**	0.47	0.31, 0.72	**< .001**
Premedication before RDD						
H1‐antihistamine	2.95	1.28, 6.76	.**011**	1.29	0.35, 4.79	.701
H2‐antihistamine	2.31	1.03, 5.19	.**042**	2.17	0.59, 7.97	.244
Leukotriene receptor antagonist	3.73	1.24, 11.25	.**019**	2.10	0.51, 8.70	.306

*Note*: Bolded values indicate statistically significant differences between groups.

Abbreviation: HSR, hypersensitivity reactions; RDD, rapid drug desensitization.

## 4 DISCUSSION

This study showed that RDD remains an effective and generally safe strategy for pediatric hemato‐oncology patients who experience immediate HSRs to chemotherapeutic agents. In our cohort of 38 children undergoing 182 RDDs, BTRs occurred in 29 procedures (15.9%). Although most BTRs were mild and two‐thirds of affected procedures were ultimately completed after on‐site treatment. Furthermore, we identified additional independent risk factors associated with BTRs included the use of *E. coli*‐Asparaginase during RDD, a higher initial starting dose (lower starting ratio), and a fewer desensitization cycles. Interestingly, asparaginase, vincristine, cyclophosphamide, platinum‐base agents, and doxorubicin were frequently associated with Type I HSRs, while methotrexate, cyclosporin A and etoposide were connected to both Type I HSRs and either IRRs or CRRs.

In our cohort of pediatric hemato‐oncology patients, BTRs occurred in 15.9% of RDD procedures. Compared with prior pediatric reports (which generally report BTR rates in the lower range of ~9–13% in mixed cohorts),^6^, ^19^ our observed 15.9% rate is at the higher end of published pediatric series and lower than many adult cohorts (≈25%),^6^, ^14^, ^15^, ^16^, ^17^ likely reflecting differences in culprit agents (asparaginase, vinca alkaloids, and antimetabolites are more common in pediatric practice) and local protocols.

Although most BTRs were mild and two‐thirds of affected procedures were successfully completed after treatment, one‐third of BTRs resulted in termination of RDD—indicating a clinically meaningful burden. Termination was primarily driven by airway‐threatening or adrenaline‐requiring events (all five adrenaline‐treated, terminated procedures were associated with *E. coli*‐asparaginase), and by persistent or recurrent cutaneous reactions despite premedication in other cases. Thus, while RDD allows continued access to first‐line agents for many children, BTRs—especially those leading to incomplete desensitization—pose significant clinical and logistical challenges (e.g., need for alternative agents, switching to Erwinia asparaginase, or changing chemotherapy regimens).

*E. coli*‐asparaginase deserves particular attention because of its strong association with incomplete RDDs and adrenaline‐requiring events in our cohort. Biologically, asparaginase is highly immunogenic and hypersensitivity may be mediated by anti‐asparaginase antibodies that promote immune complex formation, complement activation, or enhanced mast‐cell/basophil activation; both IgE‐dependent and non‐IgE pathways have been implicated in prior reports.^5^, ^24^ These mechanisms could explain the frequent type‐I–pattern reactions and the propensity for severe, early‐cycle BTRs we observed. However, we did not measure anti‐drug antibodies, specific IgE, or other immunologic biomarkers systematically in this study, which limits mechanistic inference. While some series report successful desensitization with careful premedication, others document recurrent anaphylaxis despite intervention.^25^, ^26^, ^27^ When available, substitution to Erwinia‐ or PEG‐asparaginase is preferred;^28^, ^29^ where substitution is not feasible, adjusted RDD protocols (tailored premedication and cautious starting doses) are pragmatic alternatives.

Another key finding was that initiating RDD at a lower starting ratio (≤1:10,000 of the total dose—i.e., a higher initial dose) was independently associated with increased BTR risk. International guideline typically recommend starting ratios ranging from 1:10,000 to 1,000,000—or in select cases even 1:100 to 1:1—based on individual risk assessment.^11^, ^13^, ^30^, ^31^ A recent study by Sala‐Cunill A et al.^32^, ^33^ reported that one‐bag desensitization protocols yielded outcomes equivalent to those of three‐bag protocol in adult patients receiving chemotherapeutic and biological agents.^32^, ^33^ Additionally, Madrigal‐Burgaleta R et al.^6^ demonstrated that the one‐bag protocol for cetuximab was both effective and safe, with results comparable to those observed for other chemotherapeutic drugs that utilize three‐bag dilutions. In our cohort we found no independent association between the nominal number of bags used and BTR occurrence. Several explanations likely account for this. First, limited variability in bag formats (most procedures used multibag regimens) reduced statistical power to detect differences by bag count. Second, bag number and starting ratio are partially collinear; the multivariable model retained the more directly meaningful parameter (starting ratio). Third, protocols were individualized in practice—clinicians adjusted step timing, bag volumes, escalation speed, and premedication—so bag count alone does not capture the true initial exposure or escalation profile. We therefore emphasize starting ratio/initial dose as the more informative procedural variable in our dataset and acknowledge that the lack of standardized, detailed protocol documentation limits mechanistic interpretation.

Our results show an inverse association between desensitization cycle number and BTR (adjusted OR 0.47 per additional cycle): procedures repeated over additional cycles were less likely to produce BTRs. This finding aligns with prior reports—Sala‐Cunill A et al.^32^ found approximately 50% of BTRs occurred during the first RDD with declining rates thereafter and Madrigal‐Burgaleta R et al.^6^ observed 39.2% of BTRs in the first RDD versus 11.8% in the second, irrespective of culprit drug. The protective effect of additional cycles likely reflects both acquired biological tolerance from repeated controlled exposures and protocol adjustments made after initial reactions (e.g., slower escalation, modified premedication). Clinically, these results support cautious initial dosing and consideration of stepwise escalation or planned repeat desensitization cycles to enhance safety and success rates—particularly for high‐risk agents such as *E. coli*‐asparaginase.

H1 antihistamines, H2 antihistamines, and leukotriene receptor antagonists were used more frequently in patients with BTRs compared to those without. However, after adjustment for other potential confounding factors, no statistically significant differences were found for any premedication in our study. Previous studies have shown conflicting findings, with some suggesting that systemic corticosteroids may be protective,^15^, ^25^, ^27^ while others have identified them as potential risk factors.^6^ Current clinical practice guidelines^4^, ^5^, ^13^ offer limited recommendations regarding premedication strategies for RDDs, largely due to a lack of robust evidence. Clinicians must carefully weigh the potential benefits against the risk of masking early signs of HSRs, particularly mucocutaneous manifestations.^6^, ^13^, ^30^

Serum tryptase may help distinguish HSRs from other conditions. In our cohort, none of the 11 children had an acute serum tryptase >11.4 ng/mL, but three showed a rise that met the international consensus criterion for significant increase [(acute) ≥ 1.2 × baseline +2 ng/mL]. This finding suggests that single acute measurements have limited sensitivity and that paired sampling (acute and baseline) is more informative. Paired tryptase measurements can also aid in screening for systemic mastocytosis or mast cell activation syndrome when baseline levels are elevated.^34^, ^35^

According to the EAACI Position on HSRs to chemotherapy,^5^ our understanding of the exact immunopathogenic mechanisms underlying HSRs for each drug category remains limited. Asparaginase, epipodophyllotoxins (such as etoposide), and platinum‐based agents can involve both IgE‐mediated and non‐IgE‐mediated HSRs, such as those mediated by IgG and complement activation. Additionally, epipodophyllotoxins and platinum‐based agents may trigger direct mast cell activation in some cases. However, the mechanisms associated with Vinca alkaloids (vincristine), alkylating agents (cyclophosphamide), anthracyclines, and other antitumor antibiotics (such as doxorubicin and bleomycin) remain unclear.

Our study also found that the majority of HSRs to asparaginase and platinum‐based agents occurred through Type I HSRs, consistent with previous reports. Furthermore, we identified that vincristine, cyclophosphamide, and doxorubicin were frequently associated with Type I HSRs, while etoposide, methotrexate, and cyclosporin A were linked to both Type I HSRs and either IRRs or CRRs. This classification may assist in a deeper understanding of the characteristics of HSRs associated with each chemotherapeutic agent and guide risk‐based stratification for the design of RDD protocols.

This study has several limitations. It was a single‐center study, and most participants were identified through retrospective chart reviews. In addition, a standardized RDD protocol was not applied across all patients because clinicians made individualized, risk‐based adjustments per practice guidelines; thus, heterogeneity limits causal inference. Phenotyping and endotyping were based on initial clinical history and paired serum tryptase in some cases; we did not measure IL‐6 or systematically assess anti‐drug antibodies, which would better characterize CRRs and the immunologic mechanism.

Despite these limitations and the relative scarcity of data on RDD phenotyping and endotyping in pediatric patients, our study represents one of the largest and most comprehensive analyses focused exclusively on pediatric patients receiving chemotherapeutic desensitization. A culprit drug was confirmed in approximately half of patients, which supports the validity of the clinical HSR diagnoses and the rationale for performing RDD. Procedurally, RDD proved generally safe: most BTRs were mild and two‐thirds of affected procedures were ultimately completed after on‐site management. Nevertheless, the fact that a substantial minority of BTRs led to termination highlights a clinically meaningful burden and the potential consequences of avoiding first‐line agents—substitution may compromise oncologic outcomes in pediatric hemato‐oncology.

Future large, prospective multicenter studies using standardized protocols and systematic immunologic testing (including anti‐drug antibodies, tryptase, and cytokine profiling) are needed to (1) link immunologic confirmation to RDD outcomes, (2) clarify causal factors for BTRs, and (3) define the optimal role of premedication and protocol design in pediatric RDD.

## 5 CONCLUSION

RDD is a safe and effective strategy for managing immediate HSRs—including type I HSR, CRR, and mixed reactions—in pediatric hemato‐oncology patients. BTRs were mostly mild and manageable but were associated with *E. coli*‐Asparaginase RDDs, higher initial starting dose, and early cycles. Individualized protocols, careful risk assessment, and expert supervision are essential; further studies with standardized risk stratification are required to validate and improve desensitization practice.

## AUTHOR CONTRIBUTIONS

**Kantima Kanchanapoomi:** Writing – review and editing. **Pathitta Doradee:** Data curation; writing – original draft; methodology; formal analysis; writing – review and editing; conceptualization; software. **Orathai Jirapongsananuruk:** Writing – review and editing; conceptualization. **Prapasri Kulalert:** Writing – review and editing; validation; formal analysis; software. **Witchaya Srisuwatchari:** Conceptualization; methodology; validation; supervision; writing – original draft; writing – review and editing; formal analysis; project administration; software; data curation; visualization. **Punchama Pacharn:** Writing – review and editing; conceptualization. **Supaluk Tangvalelerd:** Writing – review and editing.

## FUNDING INFORMATION

The authors have nothing to report.

## CONFLICT OF INTEREST STATEMENT

The authors declare no conflicts of interest.

## Supporting information


**Table S1.** Clinical Characteristics of hypersensitivity reactions (HSRs) and potential classification of 52 suspected initial chemotherapy HSRs.

**Table S2.** Summary of potential classification of initial hypersensitivity reactions to 52 chemotherapeutic agents.

**Table S3.** Summary of the confirmation of the culprit drug in a total of 38 patients with 52 suspected initial hypersensitivity reactions (HSRs).

## Data Availability

The data that support the findings of this study are available on request from the corresponding author. The data are not publicly available due to privacy or ethical restrictions.
