# Supplementary material for: Breakthrough reactions in pediatric chemotherapeutic desensitization: Outcomes and associated risk factors
Source: Pediatr Allergy Immunol. 2026 Jul 13;37(7):e70425. doi: 10.1111/pai.70425 (PMC13365207; doi:10.1111/pai.70425)
Supplement: Supplementary file 1 — Table S1. Clinical Characteristics of hypersensitivity reactions (HSRs) and potential classification of 52 suspected initial chemotherapy HSRs. [file PAI-37-e70425-s001.docx]

**Supplementary Table 1. Clinical Characteristics of hypersensitivity reactions (HSRs) and potential classification of 52 suspected initial chemotherapy HSRs**

| Drugs | Onset of reaction | First infusion | Associated symptoms | Initial reaction severity grading | Treatment during initial reaction | Confirmed culprit drugs | TA  (ng/mL) | TB  (ng/mL) | Any BTR | Possible classification | Remarks |
| --- | --- | --- | --- | --- | --- | --- | --- | --- | --- | --- | --- |
| Cyclosporin | <1h | Yes | No | 2 | Adrenaline/  systemic steroid | N/A | 3.20 | 2.35 | Yes | CRR | At first infusion, not responding to premedication |
| Methotrexate | 1-6h | Yes | Fever | 1 | AH1/AH2 | N/A | N/A | N/A | Yes | CRR | At first infusion, associted with fever, not responding to premedication |
| Asparaginase | 1-6h | Yes | No | 2 | AH1 | N/A | N/A | N/A | Yes | CRR | At first infusion, not responding to premedication |
| Mesna | <1h | Yes | No | 1 | AH1 | N/A | N/A | N/A | Yes | CRR | At first infusion, not responding to premedication |
| Carboplatin | <1h | Yes | No | 1 | AH1 | Neg | N/A | N/A | No | IRR | At first infusion, subside gradually with the following infusion |
| Etoposide | <1h | Yes | No | 1 | AH1 | N/A | N/A | N/A | No | IRR | At first infusion, subside gradually with the following infusion |
| Cyclophosphamide | <1h | Yes | No | 1 | AH1 | N/A | N/A | N/A | No | IRR | At first infusion, subside gradually with the following |
| Cyclosporin | <1h | Yes | No | 3 | Adrenaline | Neg | 1.72 | <1.0 | No | IRR | At first infusion, subside gradually with the following infusion |
| Methotrexate | <1h | Yes | No | 1 | Adrenaline/AH1/AH2 | N/A | N/A | N/A | No | IRR | At first infusion, subside gradually with the following infusion |
| Etoposide | <1h | Yes | No | 2 | Adrenaline/AH1 | Neg | 1.24 | 1.21 | No | IRR | At first infusion, subside gradually with the following infusion |
| Etoposide | <1h | No | No | 1 | AH1 | N/A | N/A | N/A | No | Type I | At repeated infusion |
| Asparaginase | <1h | No | No | 2 | Adrenaline/AH1 | DPT | N/A | N/A | Yes | Type I | At repeated infusion, positive DPT |
| Asparaginase | 1-6h | No | No | 1 | AH1 | SPT | N/A | N/A | Yes | Type I | At repeated infusion, positive SPT |
| Asparaginase | 1-6h | No | No | 1 | AH1/AH2 | DPT | N/A | N/A | Yes | Type I | At repeated infusion, positive DPT |
| Asparaginase | 1-6h | No | No | 3 | Adrenaline/AH1/AH2 | N/A | 5.04 | <1.0 | No | Type I | At repeated infusion, life-threatening anaphylaxis, elevated paired serum tryptase |
| Asparaginase | 1-6h | No | No | 2 | Adrenaline/AH1 | N/A | 5.58 | 1.73 | No | Type I | At repeated infusion, elevated paired serum tryptase |
| Cytarabine | <1h | No | No | 1 | AH1/AH2 | IDT | N/A | N/A | No | Type I | At repeated infusion, positive IDT |
| Etoposide | <1h | No | No | 2 | Adrenaline | N/A | N/A | N/A | No | Type I | At repeated infusion, life-threatening anaphylaxis |
| Asparaginase | <1h | No | No | 1 | AH1 | SPT | N/A | N/A | Yes | Type I | At repeated infusion, positive SPT |
| Etoposide | <1h | No | No | 2 | N/A | Neg | N/A | N/A | No | Type I | At repeated infusion |
| Asparaginase | <1h | No | No | 2 | Adrenaline/AH1 | N/A | N/A | N/A | Yes | Type I | At repeated infusion |
| Vincristine | <1h | No | No | 2 | AH1 | N/A | N/A | N/A | No | Type I | At repeated infusion |
| Asparaginase | 1-6h | No | No | 2 | Adrenaline/AH1 | N/A | 8.51 | 1.14 | Yes | Type I | At repeated infusion, elevated paired serum tryptase |
| Methotrexate | <1h | No | No | 2 | Adrenaline/AH1 | Neg | 2.8 | 2.76 | Yes | Type I | At repeated infusion |
| Carboplatin | <1h | No | No | 1 | AH1 | IDT | N/A | N/A | No | Type I | At repeated infusion, positive IDT |
| Vincristine | 1-6h | No | No | 2 | Adrenaline/AH1 | Neg | 1.06 | 1.21 | No | Type I | At repeated infusion |
| Doxorubicin | 1-6h | No | No | 2 | Adrenaline/AH1 | Neg | N/A | N/A | No | Type I | At repeated infusion |
| Cyclophosphamide | <1h | No | No | 2 | Adrenaline/AH1 | N/A | N/A | N/A | No | Type I | At repeated infusion |
| Asparaginase | <1h | No | No | 1 | AH1/AH2 | Neg | N/A | N/A | No | Type I | At repeated infusion |
| Carboplatin | <1h | No | No | 1 | AH1/AH2 | N/A | N/A | N/A | Yes | Type I | At repeated infusion |
| Cyclosporin | <1h | No | No | 1 | AH1/AH2 | N/A | N/A | N/A | Yes | Type I | At repeated infusion |
| Oxaliplatin | <1h | No | No | 1 | N/A | Neg | N/A | N/A | Yes | Type I | At repeated infusion |
| Carboplatin | <1h | No | No | 1 | AH1 | N/A | N/A | N/A | No | Type I | At repeated infusion |
| Vincristine | <1h | No | No | 1 | AH1 | Neg | N/A | N/A | No | Type I | At repeated infusion |
| Methotrexate | <1h | No | No | 2 | Adrenaline | N/A | N/A | N/A | No | Type I | At repeated infusion |
| Vincristine | <1h | No | No | 2 | Adrenaline/AH1/AH2 | Neg | N/A | N/A | No | Type I | At repeated infusion |
| Asparaginase | <1h | No | No | 2 | Adrenaline/AH1/AH2 | SPT | N/A | N/A | Yes | Type I | At repeated infusion, positive SPT |
| Vincristine | 1-6h | No | No | 1 | AH1 | DPT | N/A | N/A | No | Type I | At repeated infusion, positive DPT |
| Asparaginase | 1-6h | No | No | 1 | AH1 | DPT | N/A | N/A | No | Type I | At repeated infusion, positive DPT |
| Methotrexate | 1-6h | No | No | 1 | AH1 | N/A | N/A | N/A | Yes | Type I | At repeated infusion |
| Mesna | <1h | No | No | 2 | Adrenaline/AH1/AH2/  Systemic steroid | N/A | 2.23 | 1.70 | Yes | Type I | At repeated infusion |
| Cyclophosphamide | 1-6h | No | No | 2 | Adrenaline/AH1/AH2/  Systemic steroid | N/A | 2.23 | 1.70 | Yes | Type I | At repeated infusion |
| Doxorubicin | <1h | No | No | 2 | Adrenaline/AH1/AH2 | N/A | N/A | N/A | Yes | Type I | At repeated infusion |
| Mesna | <1h | No | No | 2 | Adrenaline/AH1/AH2 | N/A | N/A | N/A | No | Type I | At repeated infusion |
| Cyclophosphamide | 1-6h | No | No | 2 | Adrenaline/AH1/AH2 | N/A | N/A | N/A | No | Type I | At repeated infusion |
| Asparaginase | 1-6h | No | No | 1 | N/A | N/A | N/A | N/A | Yes | Type I | At repeated infusion |
| Vincristine | 1-6h | No | No | 1 | AH1 | N/A | N/A | N/A | No | Type I | At repeated infusion |
| Doxorubicin | 1-6h | No | No | 1 | AH1 | N/A | N/A | N/A | No | Type I | At repeated infusion |
| Etoposide | 1-6h | Yes | No | 3 | Adrenaline/AH1/AH2 | N/A | 8.11 | 6.67 | No | Type I/CRR | At first infusion, life-threatening anaphylaxis |
| Mesna | 1-6h | Yes | No | 3 | Adrenaline/AH1/AH2 | N/A | 8.11 | 6.67 | No | Type I/CRR | At first infusion, life-threatening anaphylaxis |
| Etoposide | 1-6h | Yes | Diaphoresis | 3 | Adrenaline/AH1 | N/A | 1.53 | 1.79 | No | Type I/CRR | At first infusion, life-threatening anaphylaxis, associted with diaphoresis, not responding to premedication |
| Bleomycin | 1-6h | Yes | Diaphoresis | 3 | Adrenaline/AH1 | N/A | 1.53 | 1.79 | No | Type I/CRR | At first infusion, life-threatening anaphylaxis, associted with diaphoresis, not responding to premedication |

Abbreviation: AH1, H1-antihistamine; AH2, H2-antihistamine; BTR, breakthrough reaction; CRR, cytokine release reactions; DPT, drug provocation test; h, hour(s); IDT, intradermal test; IRR, infusion-related reactions; Neg, negative; N/A, not applicable; SPT, skin prick test; TA, acute serum tryptase level; TB, baseline serum tryptase level; Type I (IgE/non-IgE)
