# Supplementary material for: Breakthrough reactions in pediatric chemotherapeutic desensitization: Outcomes and associated risk factors
Source: Pediatr Allergy Immunol. 2026 Jul 13;37(7):e70425. doi: 10.1111/pai.70425 (PMC13365207; doi:10.1111/pai.70425)
Supplement: Supplementary file 2 — Table S2. Summary of potential classification of initial hypersensitivity reactions to 52 chemotherapeutic agents. [file PAI-37-e70425-s002.docx]

**Supplementary Table 2. Summary of potential classification of initial hypersensitivity reactions to 52 chemotherapeutic agents**

| Drug category/Drugs | Possible classification of HSRs | | | |
| --- | --- | --- | --- | --- |
|  | **Type I** | **CRR** | **IRR** | **Type I /CRR** |
| Bacterial Enzyme |  |  |  |  |
| Asparaginase | 12 | 1 |  |  |
| Vinca Alkaloids |  |  |  |  |
| Vincristine | 6 |  |  |  |
| Antimetabolites |  |  |  |  |
| Methotrexate | 3 | 1 | 1 |  |
| Cytarabine | 1 |  |  |  |
| Epipodophyllotoxins |  |  |  |  |
| Etoposide | 3 |  | 2 | 2 |
| Alkylating agents and chemoprotective agents |  |  |  |  |
| Cyclophosphamide | 3 |  | 1 |  |
| Sodium-2 -mercaptoethanesulfonate  (Mesna) | 2 | 1 |  | 1 |
| Platinum-base agents |  |  |  |  |
| Carboplatin | 3 |  | 1 |  |
| Oxaliplatin | 1 |  |  |  |
| Calcineurin inhibitors |  |  |  |  |
| Cyclosporin A | 1 | 1 | 1 |  |
| Anthracyclines and other antitumor antibiotics |  |  |  |  |
| Doxorubicin | 3 |  |  |  |
| Bleomycin |  |  |  | 1 |

Abbreviation: HSR, hypersensitivity reactions; CRR, cytokine release reactions; IRR, infusion-related reactions; Type I (IgE/non-IgE)
