# Supplementary material for: Breakthrough reactions in pediatric chemotherapeutic desensitization: Outcomes and associated risk factors
Source: Pediatr Allergy Immunol. 2026 Jul 13;37(7):e70425. doi: 10.1111/pai.70425 (PMC13365207; doi:10.1111/pai.70425)
Supplement: Supplementary file 3 — Table S3. Summary of the confirmation of the culprit drug in a total of 38 patients with 52 suspected initial hypersensitivity reactions (HSRs). [file PAI-37-e70425-s003.docx]

**Supplementary Table 3. Summary of the confirmation of the culprit drug in a total of 38 patients with 52 suspected initial hypersensitivity reactions (HSRs).**

| Confirmation methods to identify the culprit drugs | Total (N=52) |
| --- | --- |
| Skin prick test, n (%) | 3 (5.8%) |
| Intradermal test, n (%) | 2 (3.8%) |
| Drug provocation test, n (%) | 4 (7.7%) |
| BTR, n (%) | 15 (28.8%) |
